# Seasonal dietary changes increase the abundances of savanna herbivore species

**DOI:** 10.1126/sciadv.abd2848

**Published:** 2020-10-02

**Authors:** A. Carla Staver, Gareth P. Hempson

**Affiliations:** 1 Ecology and Evolutionary Biology, Yale University, New Haven, CT 06511, USA.; 2 Centre for African Ecology, School of Animal, Plant and Environmental Sciences, University of the Witwatersrand, Johannesburg, South Africa.; 3 South African Environmental Observation Network (SAEON), Ndlovu Node, Phalaborwa Gate, Kruger National Park, South Africa.

## Abstract

African savannas are home to the world’s last great megafaunal communities, but despite ongoing population declines, we only poorly understand the constraints on savanna herbivore abundances. Seasonal diet shifts (except migration) have received little attention, despite a diversity of possible dietary strategies. Here, we first formulate two theoretical models that predict that both mixed feeding and migratory grazing increase population sizes. These predictions are borne out in comprehensive data across African savanna parks: Mixed feeders are the most abundant herbivores in Africa, alongside a few migratory grazer populations. Overall, clear mixed-feeder dominance may reflect a historical pattern or may instead mirror a general global decline in specialists. Regardless, mixed feeders dominate the savannas of the present and future.

## Teaser

African savanna herbivores that migrate or change diets seasonally are more populous than their specialist counterparts.

## INTRODUCTION

African savanna herbivores are increasingly restricted to a dwindling set of protected areas (*1*), subject to widespread savanna degradation (*2*, *3*) and to resultant intensification of the effects of drought (*4*), predators (*5*), and epidemic diseases (*6*, *7*). Together, these escalating pressures have resulted in major but poorly understood declines in ungulate population numbers (*1*). A deeper understanding of the factors that regulate herbivore populations will be critical to their effective management and persistence into the future.

One of the least understood determinants of herbivore population dynamics is their dietary strategy, despite extensive evidence that diet composition can vary widely within the guild (*8*–*11*). Broadly, savanna herbivores are grazers (eating predominantly grasses; e.g., zebra, *Equus quagga*), browsers (eating predominantly trees and shrubs; e.g., giraffes, *Giraffa camelopardalis*), and lastly, mixed feeders (eating both grasses and trees/shrubs; e.g., impalas, *Aepyceros melampus*). Mixed feeders are mostly grazers in the growing season but switch to browsing during the dry season (*9*, *12*) as the nutritional quality and quantity of grass forage declines (*13*). However, despite this breadth of possible strategies in diet composition and variability among herbivores, most synthesis work on herbivore abundance has focused on the contributions of abiotic environment (*14*, *15*), body size (*16*), predation (*5*), and spatial movement (*17*) to the dynamics of the herbivore guild.

General theoretical work has suggested that species with broader diets—generalists—can potentially achieve higher population abundances than specialists, especially in the variable environments (like savannas) that promote generalist-specialist coexistence (*18*, *19*). This prediction is consistent with the observation in diverse ecosystems that widespread generalists are often abundant (*20*), although tests of the prediction often become muddled by whether empirical patterns are an artifact of extensive anthropogenization of the biosphere (*21*). Detailed simulation work has confirmed that diet variability may promote high abundances when forage is variable (*22*, *23*), but these predictions have yet to be generalized or tested against empirical patterns of herbivore abundances in real savanna ecosystems. This delay may be due, in part, to the fact that most iconic savannas are known for their super-abundance not of generalist mixed feeders but of migratory grazers (*17*, *24*).

**Fig. 1:**
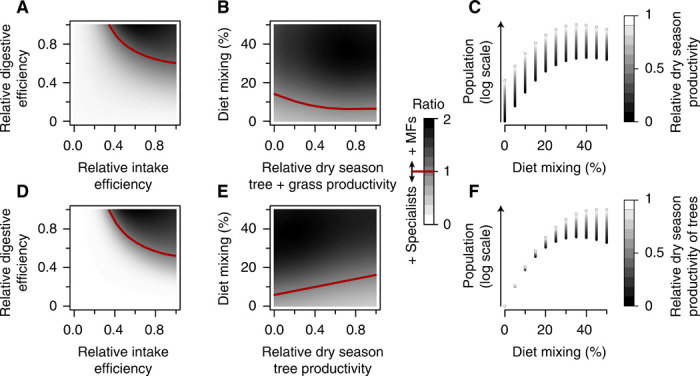
Theoretical continuous-time consumer-resource model results. Ratio of mixed feeder (MF) to specialist population sizes, depending on the relative feeding efficiency of mixed feeders (**A** and **D**) and on seasonality of productivity and the mixed-feeder diet (**B** and **E**), and the response of modeled herbivore population size as a function of annualized diet composition (**C** and **F**). Results in the top row assume that dry season productivity of both trees and grasses is 0, whereas results in the bottom row assume a less rigidly seasonal setting [with *A*_*G,*dry_ = 0.25 × *A*_*G,*wet_ and *A*_*T,*dry_ = 0.75 × *A*_*T,*wet_ in (D) and with *A*_*T,*dry_ variable in (E and F)]. In (A) and (D), the costs of mixed feeding are applied as a fractional reduction in digestive efficiency ϕ and intake efficiency β of the specialist; in (B) and (E), relative dry season productivity is calculated as a fraction of wet-season productivity; in (B), (C), (E), and (F), diet mixing is given as the overall annual diet mixing (see Materials and Methods for equation), but the mixed feeder always grazes in the wet season but switches to some fraction of browse (0 to 100%) in the dry season. Except where parameters are varied, all simulations use *A*_*G,*wet_ = 10, *A*_*T,*wet_ = 5, *K_G_* = *K_T_* = 1000, ϕ*_G_* = 0.02, ϕ*_T_* = 0.08, β*_G_* = 0.08, β*_T_* = 0.05, μ = 0.8, *c*_ϕ_ = 0.95, and *c*_β_ *=* 0.8.

**Fig. 2:**
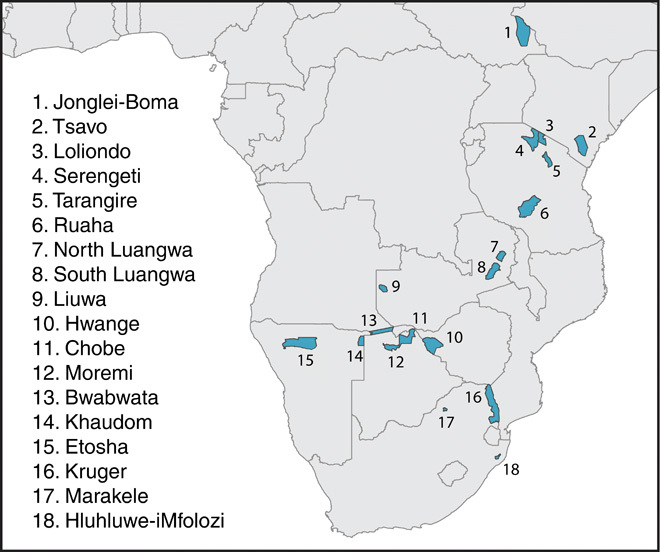
Map of African protected areas with herbivore populations included in this study, overlaid on national boundaries for reference.

Here, we ask how seasonal dietary strategies, including both mixed feeding and migration, affect herbivore populations across savanna ecosystems. We use (i) a discrete-time population model, dependent on but uncoupled to resource dynamics, and (ii) the well-studied Lotka-Volterra consumer-resource model to generate expectations for how population abundance could respond to seasonal dietary strategy, showing that reliance on a forage reservoir could increase mixed-feeder population sizes in a seasonal environment (Fig. 1 and figs. S1 to S5). We draw a theoretical analogy between migration (spatial migration between a preferred resource and a forage reservoir) and mixed feeding (a seasonal dietary migration between food types). We then test this prediction using comprehensive data (*14*) about herbivore populations across African savanna parks (see map in Fig. 2, table S1, and fig. S6 for additional species and reserve context). For more detail, see Materials and Methods.

## RESULTS AND DISCUSSION

Both theoretical models predict that mixed feeders and migratory grazers can achieve higher abundances than nonmigratory grazing or browsing specialists (Fig. 1 and figs. S1 to S5). Analysis of the discrete-time population model suggested that changing diets seasonally was broadly advantageous, especially where grasses were more seasonal than trees and where the costs of mixed feeding were low (see fig. S1). Specifically, mixed feeders achieved higher population densities than grazers and browsers when grazing was sufficiently better in the wet season, and browsing sufficiently better in the dry season, to overcome any costs of generalism (see Eq. 2). These conditions concord well with empirical observations: The feeding efficiency of mixed feeders probably is not markedly different from that of specialist grazers or browsers, and grass forage is often (although not always) more seasonally variable than tree forage.

Analysis of the coupled consumer-resource model suggested qualitatively similar results. Formally, mixed feeders were more abundant except where the costs of generalism (in digestive/energetic or intake efficiency) were high (Fig. 1, A and D, and fig. S5, A and D). System-level seasonality had little effect on the dominance of mixed feeders (Fig. 1B), but differences in seasonality of trees and grasses did (Fig. 1E); as in the discrete-time model, the coupled consumer-resource model suggested that mixed feeders were at an advantage when trees were less seasonal than grasses (Fig. 1D), although not always at an advantage against browsers when trees were truly aseasonal (Fig. 1E and fig. S5E). If anything, the advantages of seasonal dietary changes were even more robust in the coupled model; because productivity carried over from season to season, mixed feeders had access to two resource pools instead of one, irrespective of tree and grass seasonal productivity (yielding minimal differences between, e.g., fig. S5, A and D). Overall, across both models, the benefits of mixed feeding were straightforward and widespread: Mixed feeders simply had access to more and better food than specialists of either type and reached higher overall abundances.

**Fig. 3:**
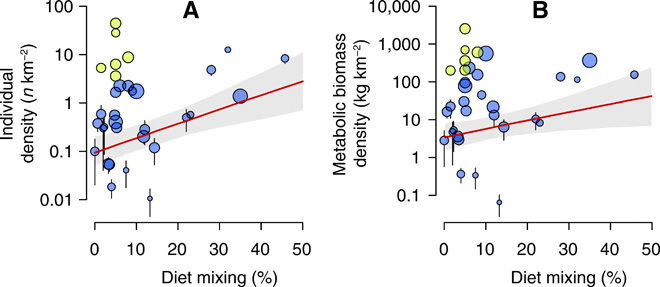
Response of herbivore population size to diet composition (where 0% = all grass or all browse and 50% = equal parts grass and browse). Annualized diet composition versus individual density (**A**) and metabolic biomass density (**B**) of common savanna ungulates in protected areas in Africa. Each point represents a species, with error bars representing standard errors of the mean across all reserves. Blue points represent nonmigratory and yellow points represent migratory populations. Point sizes scale with body mass. Best-fit statistical relationships are shown in red, although note that these relationships were fitted at the reserve and species level, not on the points actually shown (see also fig. S7; depicting all data corresponding to best fit estimates).

These predictions were borne out in reality. We observed a marked increase in herbivore abundance with increasing diet mixing (Fig. 3 and fig. S7). In the case of the density of individuals, mixed feeding alone (independent of species’ body mass; Table 1) was most predictive of herbivore abundances (Fig. 3A), in a model explaining 64.0% of variation. Meanwhile, a model including mixed feeding and body mass additively explained 72.5% of the variation in metabolic biomass density of species throughout the study area (Fig. 3B). These increases in herbivore abundance with dietary mixing suggest that seasonal dietary strategies may be a key determinant of herbivore population dynamics.

**Table 1:** Model selection results for determinants of herbivore individual density (“individual”) and metabolic biomass density (“biomass”). Species means across reserves are shown in Fig. 3 and full model results in fig. S7. These models were run on species excluding migratory populations, which we argue are subject to different constraints in that they effectively “mix” their diet by moving spatially. AIC, Akaike Information Criterion.

**Model**	**ΔAIC**
Individual ~ dietary mixing + (1 | reserve) + (1 | species)	
Individual ~ dietary mixing + body mass + (1 | reserve) + (1 | species)	1.71
Individual ~ dietary mixing × body mass + (1 | reserve) + (1 | species)	3.45
Individual ~ body mass + (1 | reserve) + (1 | species)	13.58
Biomass ~ dietary mixing + body mass + (1 | reserve) + (1 | species)	
Biomass ~ dietary mixing × body mass + (1 | reserve) + (1 | species)	1.84
Biomass ~ body mass + (1 | reserve) + (1 | species)	3.37
Biomass ~ dietary mixing + (1 | reserve) + (1 | species)	8.51

Model results for mixed feeders are directly translatable into the model predictions that migratory grazers should also have higher population sizes. This prediction, too, was realized. Migratory populations of specialist grazers also achieved unusually high individual and metabolic biomass densities (Fig. 3; note that only nonmigratory populations were included in statistical models for consistency), providing an additional line of evidence that seasonal diet strategies may be fundamental to herbivore population dynamics in African savannas. This is consistent both with a literature on migration dynamics focused on the Serengeti (*17*) and with a more general theoretical literature that draws an analogy between dietary generalists and dietary specialists that are habitat generalists (*25*). Specialists (in this case, grazers) can thus achieve high population sizes by leveraging spatial variability in resource availability.

In our statistical analyses, we have, at least explicitly, ignored major drivers of herbivore population dynamics considered fundamental elsewhere, most notably predation, which is undoubtedly the cause of extensive herbivore mortality in savannas (*5*) and also including rainfall, soils, and seasonality variation. To a degree, our statistical models implicitly capture these via random effects since random protected area effects imply common predator densities and abiotic conditions. Unfortunately, direct meta-analysis of predation effects on herbivore abundances was not possible here, because the state of predator populations in African conservation areas is poorly known. Meanwhile, although climate seasonality is relatively simple to quantify, differences in seasonality between trees and grasses (the most important aspect of seasonality for determining mixed-feeder advantages) are challenging to quantify, e.g., from remotely sensed productivity indices. However, it is also worth noting that variation within species was unexpectedly small, irrespective of predation and other random reserve effects; rather, rare species were rare everywhere and abundant ones were abundant, which suggests that commonness and rarity are determined at the species level, in part, by diet but perhaps also by other species-level traits.

Curiously, body mass was not a key determinant of individual herbivore abundance in statistical models (see Table 1), such that herbivores were equally numerous irrespective of their size. This translated to increases in herbivore biomass density with herbivore size, although this may mean little since something that has been multiplied by body mass is, of course, trivially correlated with body mass. On the face of it, it may be unexpected that small herbivores do not substantially outnumber larger ones, but there are a few plausible hypotheses to explain this result. One possibility is that higher predation rates on small herbivores flatten an underlying negative relationship between body size and individual density, effectively erasing this relationship (*5*). A second complementary possibility is that the metabolic advantages of large bodies (*16*) translate into high numbers via increased efficiency, again flattening an otherwise negative relationship. However, in view of these explanations, one might also be surprised that large-bodied species are not more numerous than they are (i.e., that individual density does not increase with body size). A final possibility is that the advantages of metabolic efficiency and predation resistance are advantageous but only in a limited way in real ecological settings, where food is limited and bulk demand becomes a disadvantage (*23*). In this scenario, perhaps elephants (*Loxodonta africana*) are locally abundant, in part, because they are mixed feeders not just because they are megaherbivores. However, historical anecdotes suggest that specialist megaherbivores (including white rhino as grazers and black rhino and giraffe as browsers) were also once abundant, suggesting that exceptionally large body size did, at one time, translate into ecological advantages. Overall, each of these possibilities is logical, and more conclusive answers will depend on models that more fully consider body size and dietary strategy effects on abundance, evaluated empirically not only against current population abundance but also against syntheses of historical population sizes of all herbivore populations.

Overall, these findings highlight the central role that dietary strategy plays in determining herbivore population dynamics in savanna ecosystems. Both mixed feeders and migratory grazers were more abundant than nonmigratory specialist grazers and browsers, in both models and reality.

The implications are fundamental. The abundant and diverse herbivore communities that make savannas so unique are, from a traditional perspective, unexpected: Why should a system with intermediate productivity be host to the peak ungulate diversity and abundance on earth (*14*)? That savannas are also the major vegetation type globally that is characterized by the codominance of two radically different plant functional types (*26*) may offer an explanation; tree-grass coexistence may contribute to facilitating the long-term persistence and diversification of herbivores, especially mixed feeders (*27*). How tree-grass interactions vary to actually shape mixed-feeder advantages will be of further interest, particularly our theoretical prediction that the difference in seasonality between grass and trees is crucial for mixed feeders to succeed. Testing this prediction may represent a real challenge, though, since differentiating between tree and grass phenology depends on field-based approaches to phenology and is not currently possible from remote sensing alone (*28*).

The preponderance of mixed feeders in savannas has implications for savanna vegetation as well. The effects of elephants on trees in savannas have been widely documented (*29*), and a smaller literature has argued that smaller-bodied mixed feeders may be equally important in limiting tree growth (*30*, *31*). Here, theoretical results show that because they attain higher population abundance, mixed feeders also more severely affect the vegetation biomass of both trees and grasses (fig. S2). From an applied perspective, wild mixed feeders (e.g., impala, *A. melampus*) in protected areas may provide a key ecosystem service by preventing more widespread woody encroachment in response to global change. The same phenomenon may help explain why grazing lawns are paradoxically tree free, if the species responsible for grazing and browsing intensely are one and the same—the mixed feeders.

Of course, herbivores can also be abundant in predominantly grassy systems, too, mostly under conditions when grazers migrate. This raises questions about the history of herbivore abundance in grassy ecosystems. Historically, migratory grazers may have been more ubiquitous, even dominant—think of the American bison, before fencing and fragmentation restricted great herbivore migrations (*24*, *32*). This would be consistent with a general global decline of specialists (*21*) and might also explain why the diversity of true grazers in African savannas is so much higher than the diversity of mixed feeders and browsers (see fig. S6) despite the fact that browsing is an older evolutionary strategy (*27*) and mixed feeding is so advantageous for maintaining large populations today.

If migratory grazers were historically dominant, the implications for savanna ecology would be far reaching. Today, experiments generally show that abundant mixed feeders can directly limit trees and shrubs in savannas from the top down (*30*), but work in the Serengeti suggests that superabundant grazers instead suppress fire regimes, releasing trees from the effects of fire (*6*). The predominant effect of herbivores on savanna vegetation structure may thus have been quite different in the past.

In sum, a fuller understanding of the processes that determine herbivore population dynamics will rely on disentangling the roles of differential seasonality of tree versus grass forage, herbivore body size, predation, and spatial versus dietary migration. Here, we have shown that seasonal dietary strategies are of the first importance in regulating savanna herbivore abundances and deserve equal consideration alongside processes that have historically received more attention. The result will be an improved understanding of how to manage and conserve increasingly threatened herbivore populations and of the ecology of savannas more broadly.

## MATERIALS AND METHODS

For theoretical intuition into the effects of different dietary strategies on herbivore population dynamics, we combine the analysis of two models: one extremely simple discrete-time population model that does not explicitly consider vegetation pools but assumes seasonal variation in forage quality and another that adapts the well-studied Lotka-Volterra consumer-resource model with one herbivore and two logistically growing resource pools, corresponding either to grass and trees or two different grass pools between which herbivores migrate. We are primarily interested in how herbivore population sizes change with respect to the degree of seasonal diet switching by herbivores, which depend on including limitations on plant productivity (capturing the benefit of switching diet, as either forage quality or total availability is depleted seasonally) and herbivore feeding behavior (as intake and digestive efficiency, including possible costs of the switching strategy itself). Despite its simplicity and a long history of attention, the Lotka-Volterra–type system (described in further detail below) resists full formal analysis, and we have therefore presented predictions via computation (see Fig. 1 and figs. S2 to S5); it is for this reason that we have included the even simpler model for more complete analytical intuition.

For empirical evaluation, herbivore census data were extracted from the database previously published by Hempson *et al*. (*14*). Data were included for protected areas in Eastern and Southern Africa with an area > 500 km^2^, rainfall between 400 and 1000 mm year^−1^, and “good” conservation status at the time of the census (see Fig. 2 and fig. S6) (*33*). These criteria aim to identify environmentally comparable regions with intact wildlife populations and to minimize the intensive management and edge effects in small reserves. Data for migratory populations were more sparse, because these have been heavily depleted through hunting and fragmentation (*24*). We identified six migratory populations of four species, including (i) wildebeest (*Connochaetes taurinus*) and zebra (*E. quagga*) in the Serengeti, (ii) white-eared kob (*Kobus kob*) and tiang (i.e., topi or tsessebe, *Damaliscus lunatus*) in the Boma-Jonglei, (iii) wildebeest in Tarangire-Manyara, and (iv) wildebeest in Liuwa.

The percentage C4 grass component of African herbivore diets was estimated by synthesizing data from published sources (*8*, *9*, *11*, *34*, *35*) and averaging across regions and studies for each species (see table S1). This percentage was reflected about the 50% diet composition axis to estimate the degree of dietary mixing via the equation: 50 − |50 − percent C4 grass|. This has a maximum value of 50% when the diet is half C4 grass and a minimum value of 0% if C4 grass is all or none of the diet.

The main objective of our analyses was to determine whether the level of grass-browse mixing in a herbivore’s diet has an influence on its abundance. Herbivore abundance was estimated as individual density (the number of individuals km^−2^) or as metabolic biomass density (body mass^0.75^ × individual density km^−2^). These data were then log transformed before fitting linear mixed-effects models with scaled % dietary mixing, species body mass, and their interaction as fixed effects in the full model. Species identity and protected area identity were included as random effects to account for processes including climatic and edaphic limitations on productivity and top-down carnivore effects on herbivore populations. Models were fitted in R (version 3.3.3) using the lme4 package.

### Discrete-time herbivore population model, uncoupled to vegetation

First, we consider a population *N* that experiences alternating wet and dry seasons, with population growth from one wet season to the next described by the equation

$$N_{w,t+1}=w^{m}d^{12-m}N_{w,t}$$

where *w* corresponds to the wet-season growth rate of the population, *d* to its dry-season growth rate, *m* to the length of the wet season in months (this choice of unit of length is arbitrary and not important for model dynamics), and *t* to the time elapsed in years. If we nondimensionalize by *N*_0_ without loss of generality, then

$$N_{w}(t)={\left[w^{m}d^{12-m}\right]}^{t}$$ (1)

These growth rates can be adapted to describe the case of grazers, browsers, and mixed feeders. Grazers grow at rates *w_G_* and *d_G_* in the wet and dry season, respectively, and browsers at rates *w_B_* and *d_B_*.

We assume that mixed feeders graze in the wet season and browse in the dry season, consistent with empirical observations (*9*). Hence, we also make the crucial assumption that, in the wet season, the potential growth rate of a herbivore is higher on grass, whereas in the dry season, the potential growth rate of a herbivore is higher on browse (i.e., that *w_G_* > *w_B_* and *d_B_* > *d_G_*). We also assume that the intrinsic quality of grazing or browsing forage probably does not differ by herbivore type, but that there are potential costs to generalism that might contribute to the relative success of the strategy (*36*); therefore, we assume that mixed feeders suffer some inefficiency in how they grow on both grass and browse, scaling their wet and dry season growth rates as *c_G_w_G_* and *c_B_d_B_*, with *c_G_* and *c_B_* < 1. Note that higher *c* denotes higher mixed-feeder efficiency.

A mixed-feeder population *M* therefore achieves higher population numbers than a grazer *G* and a browser population *B*, respectively, when

1<M(t)G(t)=[(cGwG)m(cBdB)12−m]t[wGmdG12−m]t and1<M(t)B(t)=[(cGwG)m(cBdB)12−m]t[wBmdB12−m]t

conditions that hold if and only if

$$c_{G}^{m}c_{B}^{12-m}>{\left[\frac{d_{G}}{d_{B}}\right]}^{12-m}\;\text{and}\;c_{G}^{m}c_{B}^{12-m}>{\left[\frac{w_{B}}{w_{G}}\right]}^{m}$$ (2)

See fig. S1 for a graphical illustration of these conditions. Ecologically, they suggest the intuitive result that mixed-feeder abundances will exceed grazer abundances when dry season browse is sufficiently better than dry season graze to compensate the costs of mixed feeding. By the same token, mixed-feeder abundances will exceed browser abundances when wet-season graze is enough better than wet-season browse to compensate the costs of mixed feeding. In the case where there are no costs to a mixed-feeding strategy, these conditions reduce to our assumption that grass is better forage in the wet season and trees in the dry; but in the case where mixed feeding carries a cost, the success of the strategy is determined by how seasonal trees and grasses are relative to each other. Thus, the relative responses of trees versus grasses to seasonality are fundamental to determining the benefits of mixed feeding. In extreme cases, this is obvious: Diet switching is obviously disadvantageous when inefficiencies are overwhelming or when grass survives but there is nothing to browse in the dry season, as in heavily deciduous systems in the tropics.

This model can also be used to analogize the dynamic of a migratory grazer, with similar results. In that case, *c_G_w_G_* and *c_B_d_B_* correspond to the cost-adjusted growth rate of the migratory grazer on the wet season–preferred grass pool and the dry-season forage reservoir, respectively. Thus, a migratory grazer population grows to a larger size than its nonmigratory equivalent when the benefits of switching to the dry-season reservoir outweigh the costs of doing so. In this analogy, the costs of migrating may be energetic, rather than anatomical (as above), since migratory populations often have nonmigratory conspecifics. However, the analogy is limited by the fact that often, the benefit of migrating is that there is more (not better) food at the destination, and so the discrete-time model presented here is a poor analogy; see the coupled consumer-resource model below for a different perspective on this issue.

Note that the results presented in fig. S1 are qualitatively similar when we consider a discrete-time logistic model, especially when growth rates are slow relative to carrying capacity and identical when carrying capacity is taken to be the same across herbivore types (analysis not shown).

### Coupled herbivore-vegetation (Lotka-Volterra) model

We have used a variant of the well-studied Lotka-Volterra consumer-resource model with one herbivore and two resource pools, corresponding either to grass and trees or two different grass pools between which herbivores migrate. For this detailed model description, we describe the two resource pools as grass and tree foliar biomass; all that is required to turn this into a simple model for migration, however, would be to change the names of the resources to, e.g., two different grass pools between which herbivores migrate. Here, grass and tree foliar biomasses (the resources, *G* and *T*) accumulate logistically with some growth rate (~carbon assimilation, *A_G_* and *A_T_*) and carrying capacity (*K_G_* and *K_T_*). Herbivores eat grass for a fixed fraction of time θ*_G_* and eat trees the rest of the time (θ*_T_* = 1 − θ*_G_*), in proportion to their availability at a rate that depends on bite size (i.e., handling efficiency, β*_G_* and β*_T_*). Note that for the purposes of analysis, θ and β always occur together and could be considered as one parameter; however, we maintain the distinction between the two to preserve their biological meaning. Foliage is converted to herbivore biomass depending on how nutritious food is and how efficient digestion is (combined into one term, ϕ*_G_* and ϕ*_T_*). This yields the following system of equations

dGdT=AGG(1−GKG)−βGθGGHdTdt=ATT(1−TKT)−βTθTGHdHdt=[ϕGβGθGG+ϕTβTθTT]H−μH (3)

where μ is the mortality rate of the herbivore. For specialist herbivores (with either θ*_T_* or θ*_G_* = 1) in a nonseasonal environment, the equilibria of this system and their stability are well known. Those familiar with this model can skip two paragraphs to ★.

As a review, taking the example of a specialist grazer, *T* approaches its carrying capacity *K_T_* and does not interact with grass or the herbivore population. We are left with a two-dimensional system with zero isoclines from Eq. 3 at

H=AG(1−GKG)βGθG and G=ϕGβGθGμ (4)

respectively, and equilibria occur where these zero isoclines intersect (as illustrated, e.g., in fig. S2A). Stability is given by the Jacobian evaluated at equilibrium

J¯=[−AGG¯KG−βGθGG¯ϕGβGθGH¯0] (5)

for which the trace is always negative and the determinant is always positive, such that, according to Routh-Hurwitz’s stability criteria, any equilibrium that exists is also stable for all biologically realistic (i.e., positive) regions of parameter and state space (see also fig. S2, A and B). The example of a specialist grazer is directly analogous to a specialist browser as well (see fig. S2, I and J).

Analysis is slightly more complicated for a mixed feeder (with either θ*_T_* or θ*_G_* = 1) in a nonseasonal environment because the system is three dimensional (see figs. S3 and S4 for examples of trajectories in three-dimensional space) but nonetheless straightforward. In this case, equilibria are well defined by Eq. 3, and again, their stability is this time given by the (now) three-dimensional Jacobian

J¯=[−AGG¯KG0−βGθGG¯0−ATT¯KT−βTθTT¯ϕGβGθGH¯ϕTβTθTH¯0] (6)

In this case, Routh-Hurwitz’s criteria for stability require that the trace be negative, the determinant also negative, and the determinant greater than the product of the trace and the sum of the determinants of the dominant subminors; here again, it is straightforward to show that any equilibrium that exists is also stable for all biologically realistic (i.e., positive) regions of parameter and state space.

★ The next key component of the model is seasonal variation: We assume that seasons alternate predictably, with effects on plant productivity (via *A*) and, depending on herbivory type, on herbivore diet. We assume that grazers graze and browsers browse all year. However, mixed feeders change their diets seasonally (*9*), switching from wet-season grazing to dry-season browsing when grass resources are exhausted and/or decrease in quality. By analogy, a migratory grazer might change resource pools seasonally from a preferred resource to a forage reservoir in the dry season. This seasonal change in productivity complicates analysis, even when the herbivore is a specialist grazer or browser (see fig. S2, C, G, and K). Although we can be sure that plant and herbivore population trajectories are always moving toward the seasonal stable equilibria described above, there is no guarantee that the system reaches equilibrium within a season (and, in fact, given that ungulates usually live multiple if not many years, reaching equilibrium within a season seems unlikely). Instead, we see the emergence of cycles in plant and herbivore abundance in response to alternating seasons. These seem, for broad ranges of parameter space, to tend toward “stable” cycles, as the system moves along deterministic trajectories toward (but not always reaching) seasonal equilibria (see figs. S2 to S4).

In the trivial case where mixed feeders perform better in both wet and dry seasons than pure grazers or browsers, analysis would be simpler: Mixed feeders would achieve higher population sizes overall (*37*). However, we must make assumptions to mirror a reality that directly violates this most trivial case, and mixed feeders may not always perform better overall than grazers or browsers. Although more extensive work has been done on similarly structured aquatic systems that reach equilibrium within a season (*38*), currently available analytical tools cannot go much further than this. We proceed for further intuition via computation methods below.

In reality, mixed feeders do better than grazers only in the dry season (when grass has run out) and better than browsers only in the wet season (when grass is more abundant and/or easier to eat than browse). The best-case scenario in this is that mixed feeders do exactly as well as grazers when grazing and exactly as well as browsers when browsing. However, mixed-feeder disadvantages may be more severe if mixed feeders, as generalists, are less efficient grazers than grazing specialists and less efficient browsers than browsing specialists. There are two possible ways to include the costs of mixed feeding (and analogous costs of migrating spatially). Mixed feeders may digest foliage less efficiently, payable as a fractional decrease in digestive efficiency ϕ (where efficiency *=* 1 corresponds to no cost; applied multiplicatively to the first two terms of Eq. 3). Alternatively, mixed feeders may have less efficient mouth shapes for grazing and browsing, resulting in a decrease in intake efficiency β (applied multiplicatively to the last terms of the first two of Eq. 3 and the first two terms of the last of Eq. 3).

Via computation across a broad range of parameter space, we find that when the costs of mixed feeding are high, mixed feeders do not achieve higher abundances than grazers and browsers (see Fig. 1, A and D, and fig. S5, A and D). However, mixed-feeder advantages are relatively robust to mild decreases in feeding efficiency due to mixed feeding; in fact, both intake and digestive efficiency costs are widely debated in the literature, and recent syntheses suggest that mixed feeders have only slightly lower feeding or digestive efficiencies than grazers or browsers (*36*). Thus, we should expect mixed feeders to have increasing abundances with increases in the degree of mixed feeding, for realistic efficiency estimates (see Fig. 1, C and F).

To generate computation results, we have used Runge-Kutta fourth-order integration in the package deSolve in R, version 3.2.2. For all results shown herein, wet and dry seasons each last one-half a time step (with one unit of time assumed to be a year), and the transition between the two is abrupt (instead of, e.g., sinusoidal, which would capture a more gradual transition between wet and dry seasons). In the main text (see Fig. 1), we present results assuming *A*_*G,*wet_ = 10, *A*_*T,*wet_ = 5, *A*_*G,*dry_ = *A*_*T,*dry_ = 0, *K_G_* = *K_T_* = 1000, ϕ*_G_* = 0.02, ϕ*_T_* = 0.08, β*_G_* = 0.08, β*_T_* = 0.05, μ = 0.8, *c*_ϕ_ = 0.95, and *c*_β_ *=* 0.8, except where parameters are varied for the parameter sweep, incorporating the assumptions that tree foliage is more nutritious than grass but that taking large bites of grass is easier than selective browsing of trees (see also fig. S3 for trajectories for a subset of those simulations). However, for generality, we also provide another simulation set that makes neutral assumptions about the quality and handling times of grass and trees (*A*_*G,*wet_ = *A*_*T,*wet_ = 10, *K_G_* = *K_T_* = 1000, ϕ*_G_* = ϕ*_T_* = 0.05, β*_G_* = β*_T_* = 0.05, μ = 0.8, *c*_ϕ_ = 0.95, and *c*_β_ *=* 0.8; see figs. S4 and S5). Across all simulations, grazers graze and browsers browse all year; we additionally assume that mixed feeders exclusively graze in the wet season but that they switch to browsing in the dry (with θ_*G,*wet_ *=* θ_*T,*dry_ = 1, such that diet mixing = 50%). Where we vary the degree of diet mixing for parameter sweeps (in Fig. 1, B, C, E, and F, and fig. S5, B, C, E, and F), we achieve this by varying θ_*T,*dry_.

Note that, here, we consider only the population dynamics of a single herbivore at a time, ignoring the dynamics of the diverse food web, which have been considered in some depth elsewhere (*18*, *39*). However, results using metabarcoding approaches suggest that diverse herbivores in savannas compete minimally, so this simplification may in a narrow sense be realistic; how this niche differentiation arises in a competitive, evolutionary context, especially in view of the advantages of generalism, may be of theoretical interest. Also note that our models consider the dynamics of grass and tree accumulation separately; although these may interact (*40*), tree-grass coexistence is not the subject of this work, and so we approximate equilibrium competition via limitations on the respective carrying capacities of tree and grass foliar biomass. Elaborations on these themes may be of future interest.

## Supplementary Material

abd2848_SM.pdf

## SUPPLEMENTARY MATERIALS

Supplementary material for this article is available at http://advances.sciencemag.org/cgi/content/full/6/40/eabd2848/DC1
