# Supplementary material for: Seasonal dietary changes increase the abundances of savanna herbivore species
Source: Sci Adv. 2020 Oct 2;6(40):eabd2848. doi: 10.1126/sciadv.abd2848 (PMC7852399; doi:10.1126/sciadv.abd2848)
Supplement: abd2848_SM.pdf [file abd2848_SM.pdf]

Science Advances
AAAS

advances.sciencemag.org/cgi/content/full/6/40/eabd2848/DC1

Supplementary Materials for

Seasonal dietary changes increase the abundances of savanna herbivore species

A. Carla Staver* and Gareth P. Hempson

*Corresponding author. Email: carla.staver@yale.edu

Published 2 October 2020, *Sci. Adv.* **6**, eabd2848 (2020)
DOI: 10.1126/sciadv.abd2848

This PDF file includes:

Figs. S1 to S7
Tables S1 to S2
References

Supplementary Materials

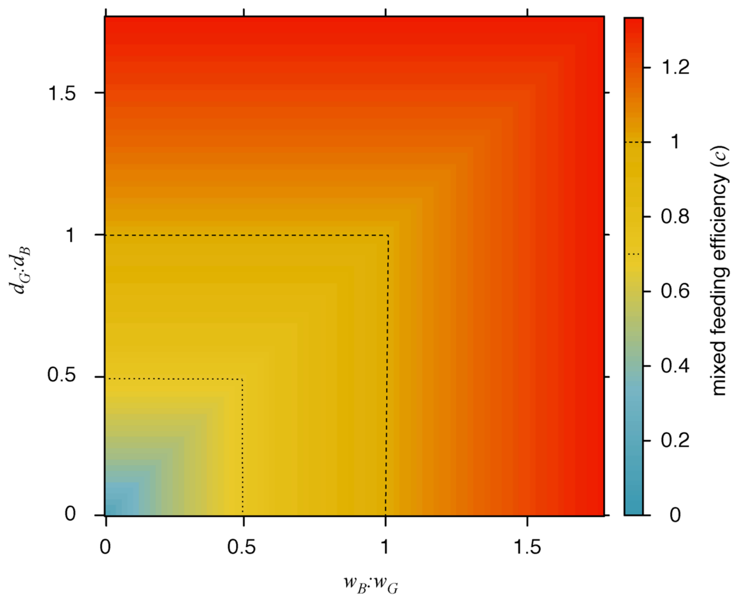

fig. S1. Results of the discrete population model. These results show the feeding efficiency (*c*) required to ensure that mixed feeder population sizes exceed both grazer and browser population sizes, depending on the ratios of population growth rates on wet-season browse vs. graze (w_B:w_G) and on dry-season graze vs. browse (d_G:d_B) (see Eq. 2 for conditions). Feeding efficiency $c > 1$ is not ecologically realistic since there are thought to be at least minimal costs to generalist strategies. However, only the lower left quadrant of the plot (delineated by a long-dash line) satisfies our assumption that grass is better forage in the wet season and trees are in the dry season; as such, for all realistic values for grass and tree forage quality, there theoretically exists some mixed feeder that can achieve higher population densities than a specialist grazer or browser, although it exists in reality depends on realistic constraints on feeding efficiency.

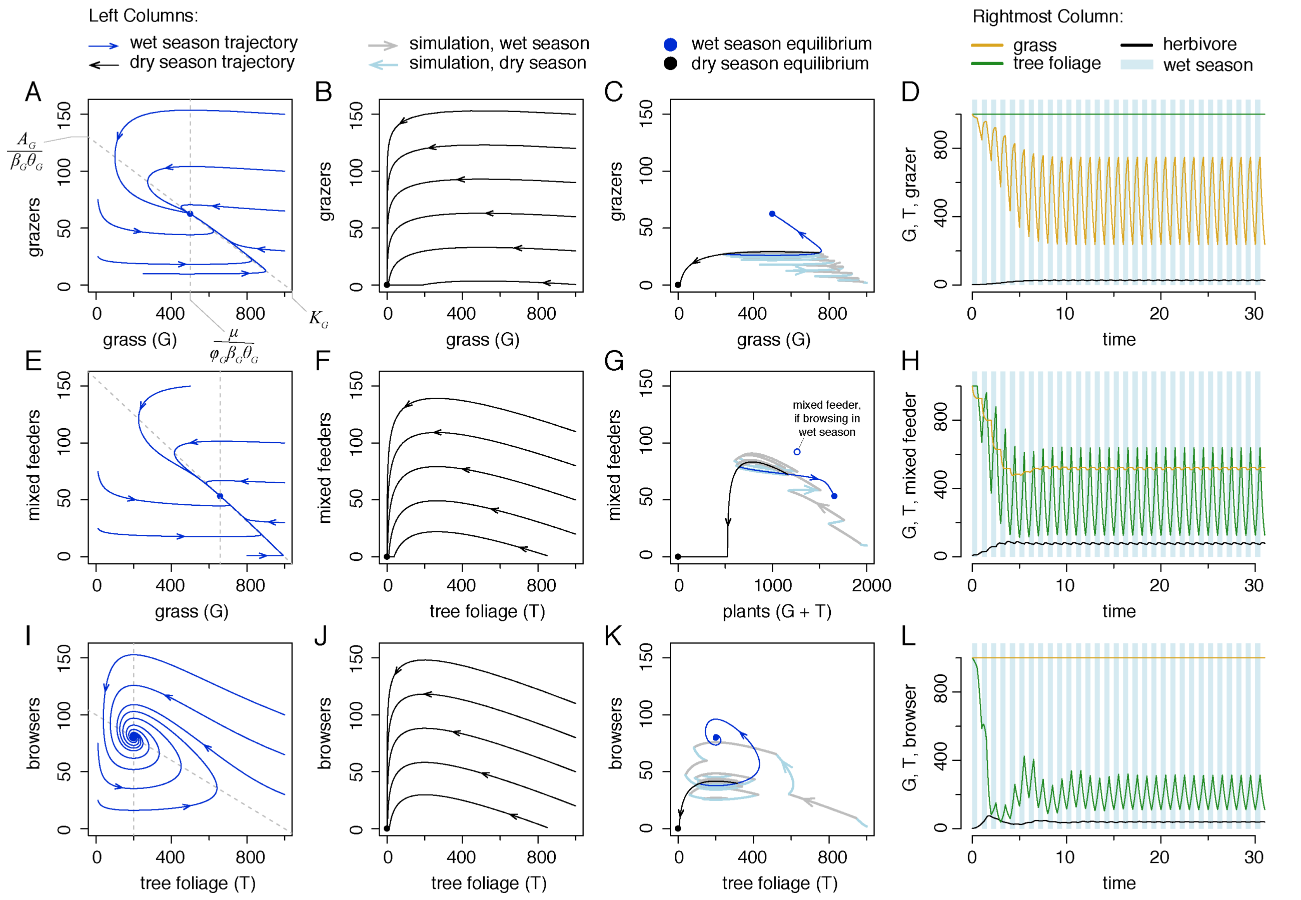

fig. S2. Coupled model phase diagrams for wet-season (A, E, I) and dry-season equilibria (B, F, J) with a grazing, a mixed-feeding, and a browsing herbivore, and simulations with alternating wet and dry seasons in state space (C, G, K) and through time (D, H, L) for each herbivore type. Parameter values correspond to those used for Fig. 1 in the main text. Except in panel G, all depictions of state space are meaningful in two dimensions; however, note that panel G represents a three-dimensional system, such that the x-axis represents the sum of two independent state variables. Simulations in panels C, G, and K were started at $G = T = 1000$ and $H = 2$.

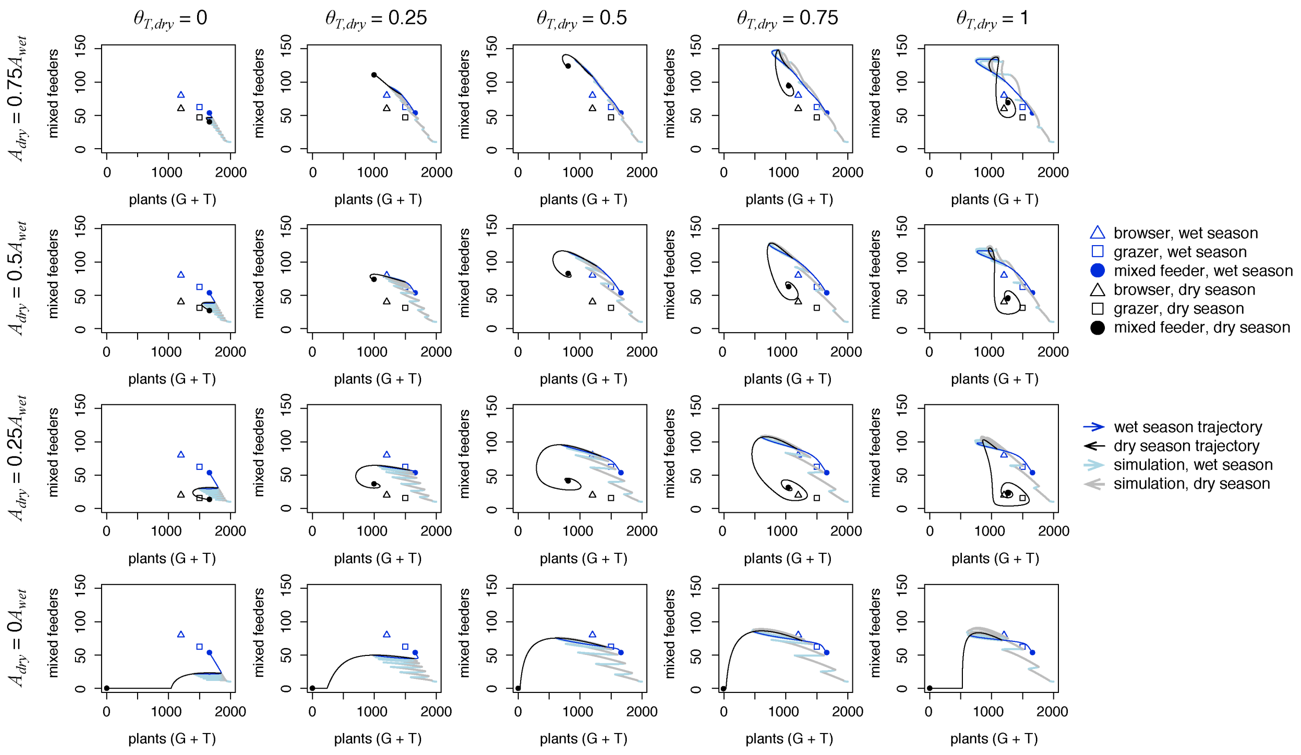

fig. S3. Coupled model simulated trajectories of mixed feeder populations through alternating wet and dry seasons (light blue and grey) and the trajectory of the system during the last year of simulation (dark blue and black), varying proportion of time spent browsing during the dry season ($\theta_{T,dry}$; from a less mixed diet on the left to more mixed on the right) and dry season productivity (from less seasonal on top to more on bottom), as a fraction of wet season productivity. These simulations show trajectories corresponding to a subset of sweep results from the top row of Fig. 1; the panel in the lower, right-hand corner corresponds to panel G from fig. S1. Note that this is not actually a two-dimensional system, such that the x-axis here represents the sum of two independent state variables; this is the reason that dry season trajectories can cross themselves (*e.g.*, on the right in the second row). All simulations with alternating seasons were started at $G = T = 1000$ and $H = 2$.

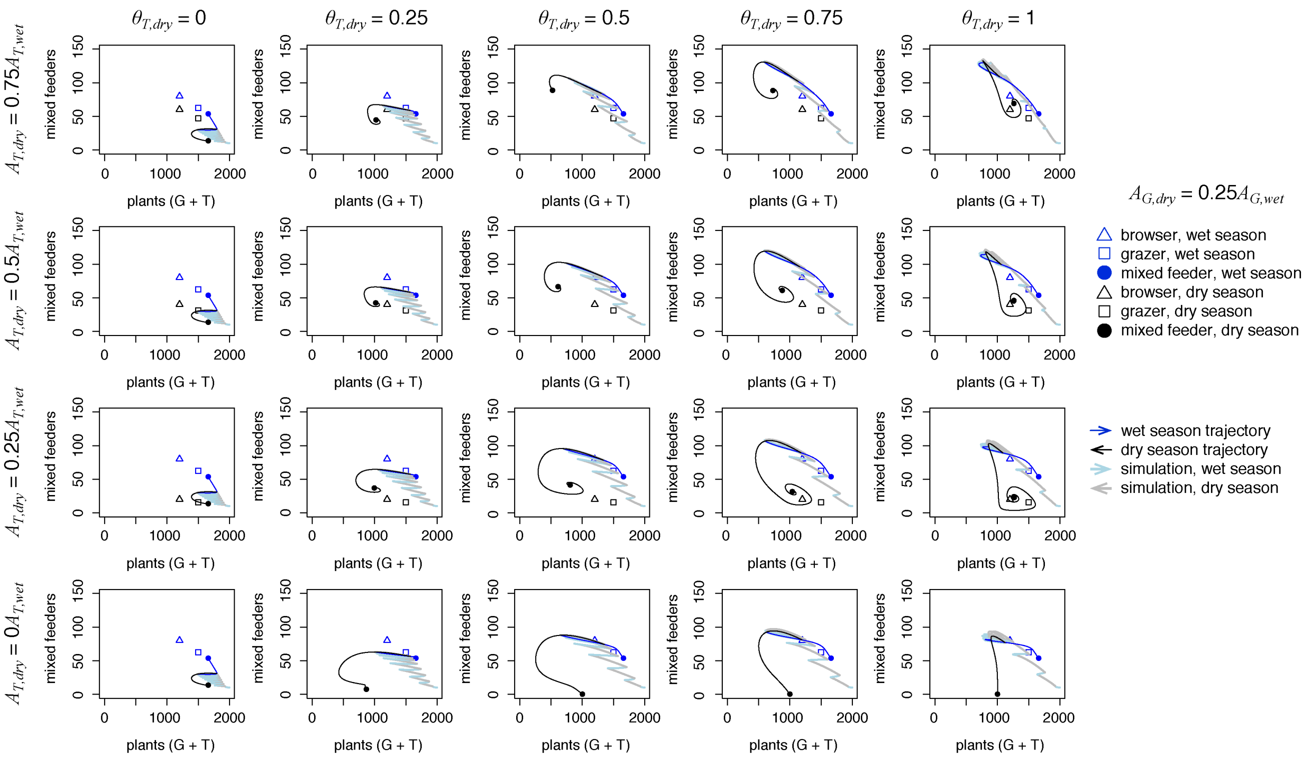

fig. S4. Coupled model simulated trajectories of mixed feeder populations through alternating wet and dry seasons (light blue and grey) and the trajectory of the system during the last year of simulation (dark blue and black), varying proportion of time spent browsing during the dry season ($\theta_{T,dry}$; from a less mixed diet on the left to more mixed on the right) and dry season tree productivity (from less seasonal on top to more on bottom), as a fraction of wet season tree productivity. Grass seasonality is fixed throughout ($A_{G,dry} = 0.25 \times A_{G,wet}$). These simulations show trajectories corresponding to a subset of sweep results from the bottom row of Fig. 1; note also that this figure differs from fig. S3 in that the seasonality of grass productivity does not vary across panels, only tree productivity. Because this is not actually a two dimensional system, the x-axis here represents the sum of two independent state variables; this is the reason that dry season trajectories can cross themselves (*e.g.*, on the right in the second row). All simulations with alternating seasons were started at $G = T = 1000$ and $H = 2$.

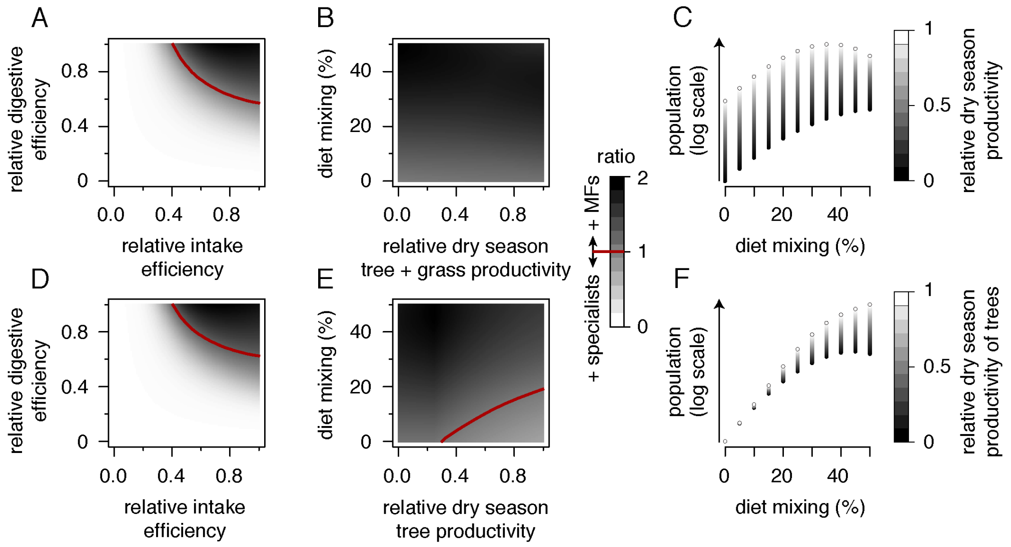

fig. S5. This figure replicates Figure 1 in the main text across a parameter set with equal trees and grass growth rates, forage quality, and handling time; except where parameters are varied, all simulations use $A_{G,wet} = A_{T,wet} = 10$, $K_G = K_T = 1000$, $\phi_G = \phi_T = 0.05$, $\beta_G = \beta_T = 0.05$, $\mu = 0.8$, $c_\phi = 0.95$, $c_\beta = 0.8$. Results in the top row assume that dry season productivity of both trees and grasses is 0, whereas results in the bottom row assume a less rigidly seasonal setting (with $A_{G,dry} = 0.25 \times A_{G,wet}$ and $A_{T,dry} = 0.75 \times A_{T,wet}$ in D and with $A_{T,dry}$ variable in E and F). In detail: Ratio of mixed feeder (MF) to specialist population sizes, depending on the relative feeding efficiency of mixed feeders (A, D) and on seasonality of productivity and the mixed feeder diet (B, E); and the response of modeled herbivore population size as a function of annualized diet composition (C, F). In A and D, the costs of mixed feeding are applied as a fractional reduction in ϕ and β of the specialist; in B and E, relative dry season productivity is calculated as a fraction of wet season productivity; in B, C, E, and F, diet mixing is given as the overall annual diet mixing (see Materials and Methods for equation), with the mixed feeder always grazing in the wet season and switching to some fraction of browse (0 to 100%) in the dry season.

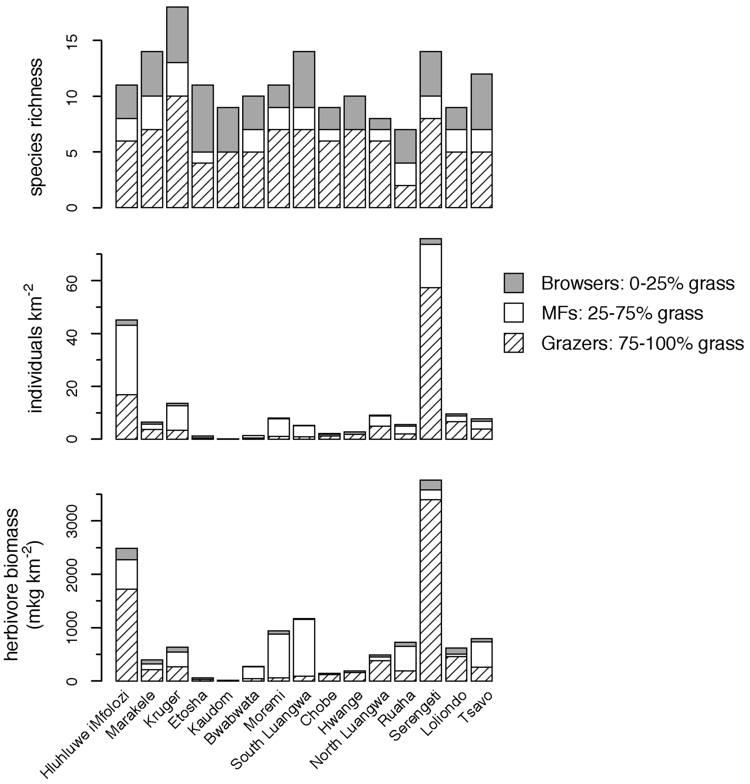

fig. S6. Herbivore species richness (top), individual density (middle), and metabolic biomass density (bottom) by dietary type across all reserves included in this study. Note that these data reflect only species for which actual abundance data are available at the reserve level, which is a variable species subset across reserves, and as such do not represent comparable totals across reserves. Because large animals are more likely to be counted, biomass totals are most comparable; species richness estimates should not be considered representative or comparable across reserves and are provided only for methodological insight.

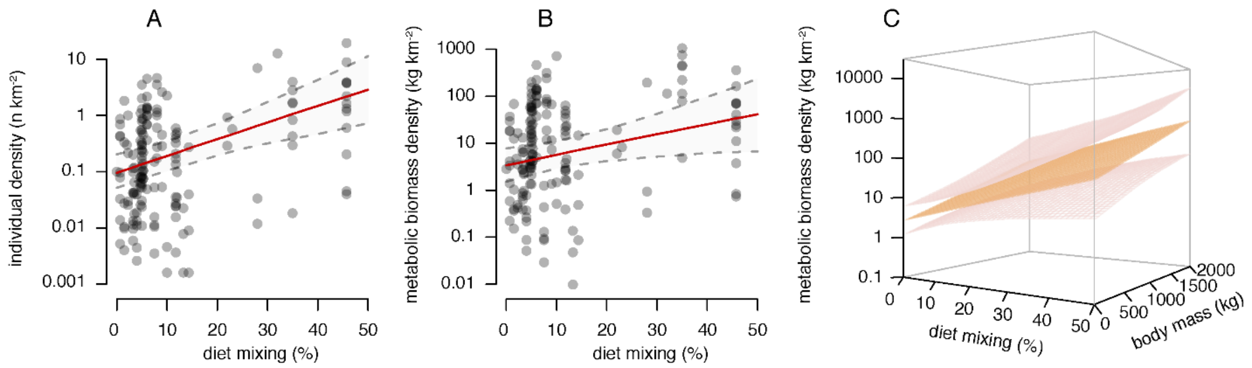

fig. S7. Annualized diet composition vs. individual density (A) and metabolic biomass density (B, C) of non-migratory savanna herbivore populations in protected areas in Africa. Each point in A and B represents a species in a reserve, with best fit relationships in red estimated from linear mixed effects models and 95% confidence intervals in grey dash estimated via bootstrapping. Panel C depicts the two-dimensional best-fit relationship that predicts metabolic biomass density, with best fit in orange and boostrapped 95% confidence intervals in pink. Bootstrapping was performed in the package bootMER in R. See Table 1 for model selection statistics.

table S1. Regional differences in percentage grass in African savanna herbivore diets. Dietary composition was determined based on stable carbon isotope evidence for proportions of C3 (browse) to C4 (grass) in faeces (*11*, *34*) and tooth enamel, bone collagen, and hair (*10*, *13*). Sources are given as column headings.

Scientific name	Common name	South Africa			East Africa			Mean C4 in diet	Mean density ± std. error
		(*11*)	(*13*)	Mean	(*10*)	(*13*)	Mean		
Aepyceros melampus	Impala	60	51	55.5	52	54	53	54.3	8.35 ± 1.83
Alcephalus buselaphus	Kongoni / Hartebeest	–	98	98	100	100	100	99.3	0.38 ± 0.12
Antidorcas marsupialis	Springbok	–	23	23	–	–	–	23.0	0.57 ± 0.03
Ceratotherium simum	White Rhinoceros	90	–	90	–	–	–	90.0	1.75 ± 0.34
Connochaetes taurinus	Common Wildebeest	90	90	90	100	100	100	95.0	1.66 ± 0.37
Damaliscus lunatus	Topi / Tsessebe	94	100	97	100	100	100	98.5	0.59 ± 0.30
Equus quagga	Plains Zebra	92	–	92	–	–	–	92.0	2.26 ± 0.41
Eudorcas thomsonii	Thomson's Gazelle	–	–	–	68	–	–	68.0	12.60
Giraffa camelopardalis	Giraffe	5	–	5	–	–	–	5.0	0.40 ± 0.07
Hippotragus equinus	Roan Antelope	96	91	93.5	100	100	100	96.8	0.05 ± 0.02
Hippotragus niger	Sable Antelope	98	100	99	94	94	94	96.5	0.05 ± 0.02
Kobus ellipsiprymnus	Waterbuck	90	100	95	92	97	94.5	94.8	0.31 ± 0.07
Litocranius walleri	Gerenuk	–	–	–	2	–	2	2.0	0.30 ± 0.26
Loxodonta africana	Savanna Elephant	35 (*34*)	–	35	–	–	–	35.0	1.38 ± 0.40
Nanger granti	Grant's Gazelle	–	–	–	22	–	22	22.0	0.50 ± 0.24
Oryx beisa	Beisa Oryx	–	–	–	88 (*35*)	–	88	88.0	0.28 ± 0.15
Oryx gazelle	Gemsbok	–	81	81	88	88	88	85.7	0.12 ± 0.07
Ourebia ourebi	Oribi	–	82	82	84	84	84	83.3	1.76 ± 0.45
Raphicerus campestris	Steenbok	7	10	8.5	18	18	18	13.3	0.01 ± 0.01
Redunca arundinum	Southern Reedbuck	–	96	96	–	–	–	96.0	0.02 ± 0.01
Sylvicapra grimmia	Common/Grey Duiker	15		15	0	–	0	7.5	0.04 ± 0.02
Syncerus caffer	Cape Buffalo	88	88	88	100	100	100	94.0	2.27 ± 0.40
Tragelaphus angasii	Nyala	23	33	28	–	–	–	28.0	4.85 ± 1.20
Tragelaphus imberbis	Lesser Kudu	–	–	–	0	–	0	0.0	0.10 ± 0.08
Tragelaphus oryx	Common Eland	3	8	5.5	18	18	18	11.8	0.20 ± 0.06
Tragelaphus scriptus	Bushbuck	9	0	4.5	0	0	0	2.3	0.32 ± 0.23
Tragelaphus strepsiceros	Greater Kudu	7	4	5.5	4	4	4	4.8	0.56 ± 0.13

table S2. Raw data for empirical analysis. Density is given as the number of individuals per km^2.

Reserve	Species	Density	Migratory	References
Bwabwata	*Aepyceros melampus*	0.040	No	52
Bwabwata	*Damaliscus lunatus*	0.004	No	52
Bwabwata	*Giraffa camelopardalis*	0.011	No	52
Bwabwata	*Hippotragus equinus*	0.010	No	52
Bwabwata	*Hippotragus niger*	0.065	No	52
Bwabwata	*Loxodonta africana*	0.830	No	52
Bwabwata	*Redunca arundinum*	0.013	No	52
Bwabwata	*Syncerus caffer*	0.362	No	52
Bwabwata	*Tragelaphus oryx*	0.027	No	52
Bwabwata	*Tragelaphus strepsiceros*	0.079	No	52
Chobe	*Aepyceros melampus*	0.203	No	41
Chobe	*Ceratotherium simum*	0.002	No	41
Chobe	*Connochaetes taurinus*	0.024	No	41
Chobe	*Equus quagga*	0.173	No	41
Chobe	*Giraffa camelopardalis*	0.079	No	41
Chobe	*Hippotragus equinus*	0.042	No	41
Chobe	*Hippotragus niger*	0.043	No	41
Chobe	*Loxodonta africana*	3.949	No	41
Chobe	*Oryx gazella*	0.002	No	41
Chobe	*Raphicerus campestris*	0.002	No	41
Chobe	*Sylvicapra grimmia*	0.004	No	41
Chobe	*Syncerus caffer*	0.694	No	41
Chobe	*Tragelaphus oryx*	0.024	No	41
Chobe	*Tragelaphus strepsiceros*	0.025	No	41
Etosha	*Aepyceros melampus*	0.045	No	48
Etosha	*Alcephalus buselaphus*	0.033	No	46,48
Etosha	*Antidorcas marsupialis*	0.570	No	46,48
Etosha	*Connochaetes taurinus*	0.159	No	46,48
Etosha	*Giraffa camelopardalis*	0.083	No	48
Etosha	*Hippotragus equinus*	0.013	No	46
Etosha	*Oryx gazella*	0.273	No	46,48
Etosha	*Raphicerus campestris*	0.022	No	46
Etosha	*Sylvicapra grimmia*	0.011	No	46
Etosha	*Tragelaphus oryx*	0.036	No	46,48
Etosha	*Tragelaphus strepsiceros*	0.063	No	46,48
Hluhluwe-iMfolozi	*Aepyceros melampus*	19.371	No	44,46
Hluhluwe-iMfolozi	*Ceratotherium simum*	2.247	No	44
Hluhluwe-iMfolozi	*Connochaetes taurinus*	3.524	No	44,46
Hluhluwe-iMfolozi	*Equus quagga*	3.527	No	44
Hluhluwe-iMfolozi	*Giraffa camelopardalis*	0.781	No	44
Hluhluwe-iMfolozi	*Kobus ellipsiprymnus*	0.457	No	44,46
Hluhluwe-iMfolozi	*Phacochoerus africanus*	2.608	No	44

Hluhluwe-iMfolozi	*Sylvicapra grimmia*	0.016	No	46
Hluhluwe-iMfolozi	*Syncerus caffer*	4.488	No	44,46
Hluhluwe-iMfolozi	*Tragelaphus angasii*	6.923	No	44
Hluhluwe-iMfolozi	*Tragelaphus strepsiceros*	1.095	No	44
Hwange	*Aepyceros melampus*	0.602	No	46
Hwange	*Connochaetes taurinus*	0.188	No	46
Hwange	*Damaliscus lunatus*	0.010	No	46
Hwange	*Kobus ellipsiprymnus*	0.075	No	46
Hwange	*Oryx gazella*	0.009	No	46
Hwange	*Redunca arundinum*	0.019	No	46
Hwange	*Sylvicapra grimmia*	0.150	No	46
Hwange	*Syncerus caffer*	0.977	No	46
Hwange	*Tragelaphus oryx*	0.135	No	46
Jonglei-Boma	*Damaliscus lunatus*	5.294	Yes	48
Jonglei-Boma	*Kobus kob*	28.000	Yes	48
Khaudom	*Alcephalus buselaphus*	0.005	No	48
Khaudom	*Connochaetes taurinus*	0.052	No	48
Khaudom	*Damaliscus lunatus*	0.010	No	48
Khaudom	*Hippotragus equinus*	0.042	No	48
Khaudom	*Raphicerus campestris*	0.008	No	48
Khaudom	*Redunca arundinum*	0.003	No	48
Khaudom	*Sylvicapra grimmia*	0.010	No	48
Khaudom	*Tragelaphus oryx*	0.004	No	48
Khaudom	*Tragelaphus strepsiceros*	0.057	No	48
Kruger	*Aepyceros melampus*	8.862	No	42,46,53
Kruger	*Alcephalus buselaphus*	0.014	No	42
Kruger	*Ceratotherium simum*	0.005	No	53
Kruger	*Connochaetes taurinus*	0.557	No	42,46,53
Kruger	*Damaliscus lunatus*	0.027	No	42,46,53
Kruger	*Equus quagga*	1.129	No	42,53
Kruger	*Giraffa camelopardalis*	0.296	No	42,53
Kruger	*Hippotragus equinus*	0.011	No	42,53
Kruger	*Hippotragus niger*	0.040	No	42,53
Kruger	*Kobus ellipsiprymnus*	0.220	No	42,46,53
Kruger	*Loxodonta africana*	0.426	No	42,53
Kruger	*Redunca arundinum*	0.039	No	42,53
Kruger	*Sylvicapra grimmia*	0.052	No	46
Kruger	*Syncerus caffer*	1.303	No	42,46,53
Kruger	*Tragelaphus angasii*	0.033	No	2,53
Kruger	*Tragelaphus oryx*	0.025	No	42,46,53
Kruger	*Tragelaphus scriptus*	0.025	No	42
Kruger	*Tragelaphus strepsiceros*	0.543	No	42,53
Liuwa	*Connochaetes taurinus*	6.276	Yes	45
Loliondo	*Aepyceros melampus*	2.194	No	49

Loliondo	*Alcephalus buselaphus*	0.423	No	49
Loliondo	*Connochaetes taurinus*	0.871	No	49
Loliondo	*Equus quagga*	4.611	No	49
Loliondo	*Giraffa camelopardalis*	0.330	No	49
Loliondo	*Loxodonta africana*	0.018	No	49
Loliondo	*Oryx beisa*	0.035	No	49
Loliondo	*Syncerus caffer*	0.683	No	49
Loliondo	*Tragelaphus oryx*	0.459	No	49
Marakele	*Aepyceros melampus*	1.616	No	42
Marakele	*Alcephalus buselaphus*	0.079	No	42
Marakele	*Connochaetes taurinus*	2.011	No	42
Marakele	*Equus quagga*	1.005	No	42
Marakele	*Giraffa camelopardalis*	0.099	No	42
Marakele	*Hippotragus equinus*	0.006	No	42
Marakele	*Kobus ellipsiprymnus*	0.138	No	42
Marakele	*Loxodonta africana*	0.296	No	42
Marakele	*Oryx gazella*	0.039	No	42
Marakele	*Phacochoerus africanus*	0.453	No	42
Marakele	*Tragelaphus angasii*	0.012	No	42
Marakele	*Tragelaphus oryx*	0.394	No	42
Marakele	*Tragelaphus scriptus*	0.030	No	42
Marakele	*Tragelaphus strepsiceros*	0.355	No	42
Moremi	*Aepyceros melampus*	3.815	No	41
Moremi	*Connochaetes taurinus*	0.235	No	41
Moremi	*Damaliscus lunatus*	0.333	No	41
Moremi	*Equus quagga*	0.275	No	41
Moremi	*Giraffa camelopardalis*	0.302	No	41
Moremi	*Kobus ellipsiprymnus*	0.075	No	41
Moremi	*Loxodonta africana*	2.816	No	41
Moremi	*Phacochoerus africanus*	0.053	No	41
Moremi	*Redunca arundinum*	0.004	No	41
Moremi	*Syncerus caffer*	0.049	No	41
Moremi	*Tragelaphus strepsiceros*	0.053	No	41
North Luangwa	*Aepyceros melampus*	3.883	No	48
North Luangwa	*Alcephalus buselaphus*	0.431	No	48
North Luangwa	*Connochaetes taurinus*	1.294	No	48
North Luangwa	*Hippotragus equinus*	0.114	No	48
North Luangwa	*Hippotragus niger*	0.118	No	48
North Luangwa	*Kobus ellipsiprymnus*	0.863	No	48
North Luangwa	*Syncerus caffer*	2.157	No	48
North Luangwa	*Tragelaphus oryx*	0.324	No	48
Ruaha	*Aepyceros melampus*	1.323	No	47
Ruaha	*Equus quagga*	0.603	No	47
Ruaha	*Giraffa camelopardalis*	0.243	No	47

Ruaha	*Loxodonta africana*	1.636	No	47
Ruaha	*Syncerus caffer*	1.397	No	47
Ruaha	*Tragelaphus oryx*	0.208	No	47
Ruaha	*Tragelaphus strepsiceros*	0.080	No	47
Serengeti	*Aepyceros melampus*	3.717	No	43,46,48
Serengeti	*Alcephalus buselaphus*	0.691	No	43,46,48
Serengeti	*Connochaetes taurinus*	44.444	Yes	50
Serengeti	*Damaliscus lunatus*	1.820	No	43,46,48
Serengeti	*Equus quagga*	8.800	Yes	51
Serengeti	*Eudorcas thomsonii*	12.600	No	48
Serengeti	*Giraffa camelopardalis*	0.500	No	43,48
Serengeti	*Hippotragus equinus*	0.098	No	46
Serengeti	*Kobus ellipsiprymnus*	0.103	No	43,46,48
Serengeti	*Nanger granti*	0.922	No	48
Serengeti	*Oryx beisa*	0.098	No	46
Serengeti	*Syncerus caffer*	1.375	No	46,48
Serengeti	*Tragelaphus oryx*	0.591	No	43,46,48
Serengeti	*Tragelaphus scriptus*	0.214	No	48
South Luangwa	*Alcephalus buselaphus*	0.096	No	48
South Luangwa	*Connochaetes taurinus*	0.061	No	48
South Luangwa	*Giraffa camelopardalis*	0.030	No	48
South Luangwa	*Hippotragus equinus*	0.200	No	48
South Luangwa	*Hippotragus niger*	0.018	No	48
South Luangwa	*Kobus ellipsiprymnus*	0.105	No	48
South Luangwa	*Redunca arundinum*	0.011	No	48
South Luangwa	*Syncerus caffer*	1.290	No	48
South Luangwa	*Tragelaphus oryx*	0.015	No	48
South Luangwa	*Tragelaphus scriptus*	1.000	No	48
Tarangire	*Connochaetes taurinus*	3.564	Yes	48
Tsavo	*Aepyceros melampus*	1.180	No	54
Tsavo	*Alcephalus buselaphus*	0.865	No	54
Tsavo	*Equus quagga*	1.280	No	54
Tsavo	*Giraffa camelopardalis*	0.125	No	54
Tsavo	*Litocranius walleri*	0.300	No	54
Tsavo	*Loxodonta africana*	1.700	No	54
Tsavo	*Nanger granti*	0.288	No	54
Tsavo	*Oryx beisa*	0.500	No	54
Tsavo	*Phacochoerus africanus*	0.285	No	54
Tsavo	*Syncerus caffer*	1.000	No	54
Tsavo	*Tragelaphus imberbis*	0.100	No	54
Tsavo	*Tragelaphus oryx*	0.170	No	54

REFERENCES AND NOTES

1. W. J. Ripple, T. M. Newsome, C. Wolf, R. Dirzo, K. T. Everatt, M. Galetti, M. W. Hayward, G. I. H. Kerley, T. Levi, P. A. Lindsey, D. W. Macdonald, Y. Malhi, L. E. Painter, C. J. Sandom, J. Terborgh, B. van Valkenburgh, Collapse of the world's largest herbivores. *Sci. Adv.* **1**, e1400103 (2015).
2. N. Stevens, C. E. R. Lehmann, B. P. Murphy, G. Durigan, Savanna woody encroachment is widespread across three continents. *Global Change Biol.* **23**, 235–244 (2016).
3. J. W. Veldman, E. Buisson, G. Durigan, G. W. Fernandes, S. le Stradic, G. Mahy, D. Negreiros, G. E. Overbeck, R. G. Veldman, N. P. Zaloumis, F. E. Putz, W. J. Bond, Toward an old-growth concept for grasslands, savannas, and woodlands. *Front. Ecol. Environ.* **13**, 154–162 (2015).
4. D. J. Augustine, Response of native ungulates to drought in semi-arid Kenyan rangeland. **48**, 1009–1020 (2010).
5. A. R. E. Sinclair, S. Mduma, J. S. Brashares, Patterns of predation in a diverse predator-prey system. *Nature* **425**, 288–290 (2003).
6. R. M. Holdo, A. R. E. Sinclair, A. P. Dobson, K. L. Metzger, B. M. Bolker, M. E. Ritchie, R. D. Holt, A disease-mediated trophic cascade in the serengeti and its implications for ecosystem C. *PLOS Biol.* **7**, e1000210 (2009).
7. H. H. T. Prins, H. P. van der Jeugd, Herbivore population crashes and woodland structure in East Africa. *J. Ecol.* **81**, 305–314 (1993).
8. T. E. Cerling, J. M. Harris, B. H. Passey, Diets of East African Bovidae based on stable isotope analysis. *J. Mammal.* **84**, 456–470 (2003).
9. D. Codron, J. Codron, J. A. Lee-Thorp, M. Sponheimer, D. de Ruiter, J. Sealy, R. Grant, N. Fourie, Diets of savanna ungulates from stable carbon isotope composition of faeces. *J. Zool.* **273**, 21–29 (2007).
10. T. R. Kartzinel, P. A. Chen, T. C. Coverdale, D. L. Erickson, W. J. Kress, M. L. Kuzmina, D. I. Rubenstein, W. Wang, R. M. Pringle, DNA metabarcoding illuminates dietary niche partitioning by African large herbivores. *Proc. Natl. Acad. Sci. U.S.A.* **112**, 8019–8024 (2015).
11. M. Sponheimer, J. A. Lee-Thorp, D. J. De Ruiter, J. M. Smith, N. J. van der Merwe, K. Reed, C. C. Grant, L. K. Ayliffe, T. F. Robinson, C. Heidelberger, W. Marcus, Diets of Southern African Bovidae: Stable isotope evidence. *J. Mammal.* **84**, 471–479 (2003).

12. J. O. Abraham, G. P. Hempson, A. C. Staver, Drought-response strategies of savanna herbivores. *Ecol. Evol.* **9**, 7047–7056 (2019).
13. M. G. Murray, A. W. Illius, Vegetation modification and resource competition in grazing ungulates. *Oikos* **89**, 501–508 (2000).
14. G. P. Hempson, S. Archibald, W. J. Bond, A continent-wide assessment of the form and intensity of large mammal herbivory in Africa. *Science* **350**, 1056–1061 (2015).
15. H. Fritz, P. Duncan, On the carrying capacity for large ungulates of African savanna ecosystems. *Proc. Biol. Sci*. **256**, 77–82 (1994).
16. H. Olff, M. E. Ritchie, H. H. T. Prins, Global environmental controls of diversity in large herbivores. *Nature* **415**, 901–904 (2002).
17. J. G. C. Hopcraft, H. Olff, A. R. E. Sinclair, Herbivores, resources and risks: Alternating regulation along primary environmental gradients in savannas. *Trends Ecol. Evol*. **25**, 119–128 (2010).
18. P. A. Abrams, The prerequisites for and likelihood of generalist-specialist coexistence. *Am. Nat*. **167**, 329–342 (2006).
19. R. A. Armstrong, R. McGehee, Competitive exclusion. *Am. Nat*. **115**, 151–170 (1980).
20. H. Ter Steege, N. C. A. Pitman, D. Sabatier, C. Baraloto, R. P. Salomão, J. E. Guevara, O. L. Phillips, C. V. Castilho, W. E. Magnusson, J.-F. Molino, A. Monteagudo, P. N. Vargas, J. C. Montero, T. R. Feldpausch, E. N. Honorio Coronado, T. J. Killeen, B. Mostacedo, R. Vasquez, R. L. Assis, J. Terborgh, F. Wittmann, A. Andrade, W. F. Laurance, S. G. W. Laurance, B. S. Marimon, B.-H. Marimon Jr., I. C. Guimarães Vieira, I. L. Amaral, R. Brienen, H. Castellanos, D. C. López, J. F. Duivenvoorden, H. F. Mogollón, F. D. de Almeida Matos, N. Dávila, R. García-Villacorta, P. R. Stevenson Diaz, F. Costa, T. Emilio, C. Levis, J. Schietti, P. Souza, A. Alonso, F. Dallmeier, A. J. Duque Montoya, M. T. Fernandez Piedade, A. Araujo-Murakami, L. Arroyo, R. Gribel, P. V. A. Fine, C. A. Peres, M. Toledo, G. A. Aymard C., T. R. Baker, C. Cerón, J. Engel, T. W. Henkel, P. Maas, P. Petronelli, J. Stropp, C. E. Zartman, D. Daly, D. Neill, M. Silveira, M. R. Paredes, J. Chave, D. de Andrade Lima Filho, P. M. Jørgensen, A. Fuentes, J. Schöngart, F. C. Valverde, A. D. Fiore, E. M. Jimenez, M. C. Peñuela Mora, J. F. Phillips, G. Rivas, T. R. van Andel, P. von Hildebrand, B. Hoffman, E. L. Zent, Y. Malhi, A. Prieto, A. Rudas, A. R. Ruschell, N. Silva, V. Vos, S. Zent, A. A. Oliveira, A. C. Schutz, T. Gonzales, M. T. Nascimento, H. Ramirez-Angulo, R. Sierra, M. Tirado, M. N. Umaña Medina, G. van der Heijden, C. I. A. Vela, E. V. Torre, C. Vriesendorp, O. Wang, K. R. Young, C. Baider, H. Balslev, C. Ferreira, I. Mesones, A. Torres-

Lezama, L. E. U. Giraldo, R. Zagt, M. N. Alexiades, L. Hernandez, I. Huamantupa-Chuquimaco, W. Milliken, W. P. Cuenca, D. Pauletto, E. V. Sandoval, L. V. Gamarra, K. G. Dexter, K. Feeley, G. Lopez-Gonzalez, M. R. Silman, Hyperdominance in the Amazonian tree flora. *Science* **342**, 1243092 (2013).

21. J. Clavel, R. Julliard, V. Devictor, Worldwide decline of specialist species: Toward a global functional homogenization? *Front. Ecol. Environ.* **9**, 222–228 (2011).
22. A. W. Illius, T. G. O'Connor, Resource heterogeneity and ungulate population dynamics. *Oikos* **89**, 283–294 (2000).
23. A. W. Illius, I. J. Gordon, Modelling the nutritional ecology of ungulate herbivores: Evolution of body size and competitive interactions. *Oecologia* **89**, 428–434 (1992).
24. G. Harris, S. Thirgood, J. Hopcraft, J. Cromsight, J. Berger, Global decline in aggregated migrations of large terrestrial mammals. *Endang. Species. Res.* **7**, 55–76 (2009).
25. W. G. Wilson, P. A. Abrams, Coexistence of cycling and dispersing consumer species: Armstrong and McGehee in space. *Am. Nat.* **165**, 193–205 (2005).
26. M. Sankaran, J. Ratnam, N. P. Hanan, Tree-grass coexistence in savannas revisited—Insights from an examination of assumptions and mechanisms invoked in existing models. *Ecol. Lett.* **7**, 480–490 (2004).
27. J. L. Cantalapiedra, R. G. FitzJohn, T. S. Kuhn, M. H. Fernández, D. DeMiguel, B. Azanza, J. Morales, A. Ø. Mooers, Dietary innovations spurred the diversification of ruminants during the Caenozoic. *Proc. Biol. Sci.* **281**, 20132746 (2013).
28. C. M. Ryan, M. Williams, J. Grace, E. Woollen, C. E. R. Lehmann, Pre-rain green-up is ubiquitous across southern tropical Africa: Implications for temporal niche separation and model representation. *New Phytol*. **213**, 625–633 (2017).
29. R. Guldemond, R. Van Aarde, A meta-analysis of the impact of African elephants on savanna vegetation. *J. Wildlife Manage*. **72**, 892–899 (2008).
30. A. C. Staver, W. J. Bond, Is there a 'browse trap'? Dynamics of herbivore impacts on trees and grasses in an African savanna. *J. Ecol*. **102**, 595–602 (2014).
31. C. A. J. O'Kane, K. J. Duffy, B. R. Page, D. W. Macdonald, Heavy impact on seedlings by the impala suggests a central role in woodland dynamics. *J. Trop. Ecol.* **28**, 291–297 (2012).
32. A. P. Dobson, M. Borner, A. R. E. Sinclair, P. J. Hudson, T. M. Anderson, G. Bigurube, T. B. B. Davenport, J. Deutsch, S. M. Durant, R. D. Estes, A. B. Estes, J. Fryxell, C. Foley, M. E. Gadd, D.

Haydon, R. Holdo, R. D. Holt, K. Homewood, J. G. C. Hopcraft, R. Hilborn, G. L. K. Jambiya, M. K. Laurenson, L. Melamari, A. O. Morindat, J. O. Ogutu, G. Schaller, E. Wolanski, Road will ruin Serengeti. *Nature* **467**, 272–273 (2010).

33. R. East, *African Antelope Database 1998* (International Union for the Conservation of Nature, 1999).
34. J. Codron, J. A. Lee-Thorp, M. Sponheimer, D. Codron, R. C. Grant, D. J. de Ruiter, Elephant (*Loxodonta africana*) diets in Kruger National Park, South Africa: Spatial and landscape differences. *J. Mammal.* **87**, 27–34 (2006).
35. D. E. Wilson, R. A. Mittermeier, *Handbook of the Mammals of the World. Vol. 2. Hoofed Mammals* (Lynx Edicions, Barcelona, 2011).
36. F. J. Perez-Barberia, D. A. Elston, I. J. Gordon, A. W. Illius, The evolution of phylogenetic differences in the efficiency of digestion in ruminants. *Proc. Biol. Sci*. **271**, 1081–1090 (2004).
37. C. A. Klausmeier, Floquet theory: A useful tool for understanding nonequilibrium dynamics. *Theor. Ecol*. **1**, 153–161 (2008).
38. C. A. Klausmeier, E. Litchman, Successional dynamics in the seasonally forced diamond food web. *Am. Nat*. **180**, 1–16 (2012).
39. N. Rooney, K. McCann, G. Gellner, J. C. Moore, Structural asymmetry and the stability of diverse food webs. *Nature* **442**, 265–269 (2006).
40. E. C. February, S. I. Higgins, W. J. Bond, L. Swemmer, Influence of competition and rainfall manipulation on the growth responses of savanna trees and grasses. *Ecology* **94**, 1155–1164 (2013).
41. "Aerial census of animals in northern Botswana: dry season 2006," (Department of Wildlife and National Parks, Gaborone, 2006).
42. "South African National Parks Annual Report 2011/2012" (South African National Parks Board report, 2012; www.sanparks.org).
43. A. R. E. Sinclair, Long term monitoring of mammal populations in the Serengeti: Census of non-migratory ungulates, 1971. *Afr. J. Ecol.* **10**, 287–297 (1972).
44. D. Druce, *Walking with African Wildlife 2012 Field Report* (Earthwatch Institute report, 2012).
45. African Parks, "Aerial Survey confirms Substantial Increase in Important Indicator Species in Liuwa" (African Parks report, 2013); www.african-parks.org/newsroom/press-releases/aerial-survey-confirms-substantial-increase-Important-indicator-species-liuwa). [accessed 15 July 2020]

46. M. J. Greenacre, E. S. Vrba, Graphical display and interpretation of antelope census data in African wildlife areas, using correspondence analysis. *Ecology* **65**, 984–997 (1984).
47. M. Norton-Griffiths, The numbers and distribution of large mammals in Ruaha National Park, Tanzania. *Afr. J. Ecol.* **13**, 121–140 (1975).
48. R. East, African Antelope Database 1998. IUCN/SSC Antelope Specialist Group. (IUCN, Gland, Switzerland and Cambridge, UK, 1999).
49. R. M. Watson, A. D. Graham, I. S. C. Parker, A census of the large mammals of Loliondo Controlled Area, northern Tanzania. *Afr. J. Ecol.* **7**, 43–59 (1969).
50. S. A. Mduma, A. R. E. Sinclair, R. Hilborn, Food regulates the Serengeti wildebeest: A 40-year record. *J. Animal Ecol.* **68**, 1101–1122 (1999).
51. S. Grange, P. Duncan, J.-M. Gaillard, A. R. E. Sinclair, P. J. Gogan, C. Packer, H. Hofer, M. East, What limits the Serengeti zebra population? *Oecologia*, **140**, 523–532 (2004).
52. T. C. Rodwell, J. Tagg, M. Grobler, Wildlife resources in the Caprivi, Namibia: The results of an aerial census in 1994 and comparisons with past surveys (Research Discussion Paper 9, Directorate of Environmental Affairs, Ministry of Environment and Tourism, Windhoek, Namibia, 1995).
53. U. de V. Pienaar, P. van Wyk, N. Fairall, An aerial census of elephant and buffalo in the Kruger National Park, and the implications thereof on intended management schemes. *Koedoe* **9**, a781 (1966).
54. W. Leuthold, B. M. Leuthold, Density and biomass of ungulates in Tsavo East National Park, Kenya. *Afr. J. Ecol.* **14**, 49–58 (1976).
